# Effect of Fenugreek Extract on Testosterone Propionate-Induced Benign Prostatic Hyperplasia

**DOI:** 10.3390/ijms26031261

**Published:** 2025-01-31

**Authors:** Jeong Yoon Lee, Jiyoung Bang, Jinhak Kim, Kwang-Soo Baek, Dongchan Oh, Yoo-Hyun Lee

**Affiliations:** 1 Department of Food and Nutrition, The University of Suwon, Hwasung 445743, Republic of Korea; 109920@naver.com (J.Y.L.); jybbb1204@naver.com (J.B.); 2 R&D Division, Daehan Chemtech Co., Ltd., Gwacheon-si 13840, Republic of Korea; jhkim@dhchemtech.com (J.K.); rnd@dhchemtech.com (K.-S.B.); fpaamgi@naver.com (D.O.)

**Keywords:** fenugreek seed extract, benign prostatic hyperplasia, androgen receptor, dihydrotestosterone

## Abstract

Benign prostatic hyperplasia (BPH) is a noncancerous urinary disorder that is common in older adult men; however, its underlying mechanisms remain unclear. Fenugreek has some biological effects, including hyperglycemia regulation, immune response modulation, and anti-cancer properties; In this study, we investigated the ameliorative effects of fenugreek seed extract (Forceterone^®^ [FCT]) in a testosterone propionate (TP)-induced BPH animal model and its mechanisms in BPH-1 human prostate epithelial cells. Sprague Dawley (SD) rats were injected subcutaneously with TP (3 mg/kg) for 8 weeks to induce BPH while FCT was administered orally at 25, 50, and 100 mg/kg. In addition, BPH-1 cells were used to evaluate the inhibitory effects on cell proliferation and examine inflammatory cytokine expression. Treating rats with FCT decreased prostate weight, dihydrotestosterone (DHT) level, and proliferating cell nuclear antigen (PCNA) expression in the prostate. Furthermore, it decreased androgen receptor (AR), 5α-reductase 2, B-cell lymphoma 2 (Bcl-2), interleukin-6 (IL-6), tumor necrosis factor-alpha (TNF-α), and NF-κB expression in vitro and in vivo and increased Bcl-2-associated X protein (Bax) expression. FCT also inhibited cell proliferation dose dependently in BPH-1 cells. These findings showed the potential use of FCT as an alternative treatment for BPH.

## 1. Introduction

The prostate is an essential accessory organ involved in seminal fluid formation, which is crucial for male reproduction [1]. It can be a target for conditions such as benign prostatic hyperplasia (BPH) with aging. BPH is an obstructive disease characterized by abnormal prostate enlargement surrounding the proximal urethra, anterior to the rectum, and below the bladder. In addition, BPH leads to urinary symptoms. The prevalence of BPH is approximately 20%, exceeds 50%, and reaches 80% in 40, 50, and 70-year-old men, respectively [2]. Lower urinary tract symptoms (LUTSs) include a weak stream, dribbling, hesitancy, and nocturia due to bladder outlet obstruction, reducing quality of life [3,4].

Surgical treatment may be considered in severe BPH cases; however, most mild-to-moderate patients use non-invasive treatments, comprising a combination of medications such as alpha-1-adrenoceptor antagonists (alpha-1 blockers) and 5 alpha-reductase inhibitors [5]. Alpha-1 blockers, including terazosin and alfuzosin, which relax the prostate muscles, and phosphodiesterase type 5 inhibitors, which are vasodilators, rapidly improve LUTSs; however, their application is limited, and they are unable to provide fundamental treatment [4]. In addition, 5 alpha-reductase inhibitors, including finasteride and dutasteride, which reduce prostate size, must be taken for a minimum of six months and popularly cause adverse side effects, such as decreased libido, erectile dysfunction, and delayed ejaculation [6,7].

However, the etiology of BPH is uncertain. Androgens, including testosterone and dihydrotestosterone (DHT), play key roles in prostate growth [8]. DHT is a potent androgen with approximately 10 times greater affinity for the androgen receptor (AR) than testosterone [9]. Testosterone is converted to DHT by 5 alpha-reductase. The use of 5 alpha-reductase inhibitors to improve BPH symptoms highlights the importance of managing DHT levels during treatment. Several studies have reported elevated DHT levels and AR expression in BPH tissues [10]. Recently, studies have suggested that reduced apoptosis [11,12] and chronic inflammation [13] are associated with BPH development.

*Trigonella foenum-graecum* (Fenugreek) is an annual herb belonging to the family Fabaceae that is widely cultivated in various regions, including North Africa [14,15]. Fenugreek has long been used as a medicinal plant, as a lactation stimulant [16], and tonic agent [17]. Recent clinical studies have reported its benefit on blood glucose control in patients with type 2 diabetes [18]. Animal studies have demonstrated several effects, including cholesterol-lowering properties [18], immune response modulation [19], and anti-cancer [20] and neuroprotective effects [19]. Fenugreek seeds contain chemical constituents including alkaloids, amino acids, and saponins [14]. In this study, we investigated the effects of fenugreek seed ethanol extract (Forceterone^®^ [FCT]) on BPH through in vitro and in vivo experiments. The fenugreek seed extract used in Rao’s studies differs from the fenugreek seed extract we used in terms of the extraction method and marker compound content [21]. We conducted this study to evaluate the efficacy of FCT on BPH and to investigate its underlying mechanisms.

## 2. Results

### 2.1. Identification of Phenolic Compound in FCT

**Figure 1:**
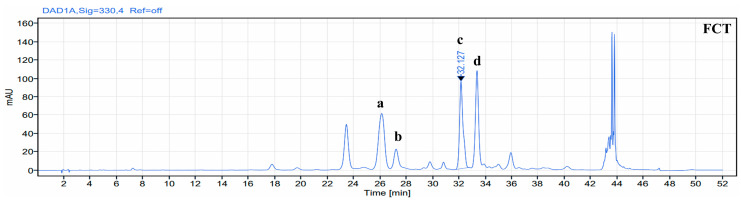
High-performance liquid chromatography analysis of standard FCT. a: iso-orientin (retention time: 26.2 min); b: orientin (retention time: 27.2); c: vitexin (retention time: 32.1 min); d: iso-vitexin (retention time: 33.1 min).

The components present in FCT were identified and represented as peaks (Figure 1). HPLC characterization showed that compounds a, b, c, and d were identified based on their respective retention times. Compound a was identified as iso-orientin, with a retention time of 26.2 min, while compound b was identified as orientin, with a retention time of 27.2 min. Compounds c and d were identified as vitexin and iso-vitexin, with retention times of 32.1 min and 33.1 min, respectively.

### 2.2. FCT Decreased Prostate Weight, DHT Levels, and 5a-Reductase 2 Expression in Rats Induced with BPH

There was no difference in body weight among the experimental animals (Supplemental Table S2). The BPH-induced group showed an 86.30% increase (*p* < 0.001) in prostate weight compared with the control group (Table 1). Administration of FCT-M (50 mg/kg) and FCT-H (100 mg/kg) resulted in significant reductions in prostate weight by 22.08 (*p* < 0.05) and 25.82% (*p* < 0.01), respectively, compared with the BPH group (Table 1). The positive control, the SAW group, had an 18.28% reduction (*p* < 0.05). The prostate ratio (prostate weight/body weight x100) in the FCT-M and FCT-H groups decreased by 22.81 and 26.91%, respectively, compared with that in the BPH group (Table 1). In contrast, the SAW group experienced a 27.76% reduction (*p* < 0.01).

**Figure 2:**
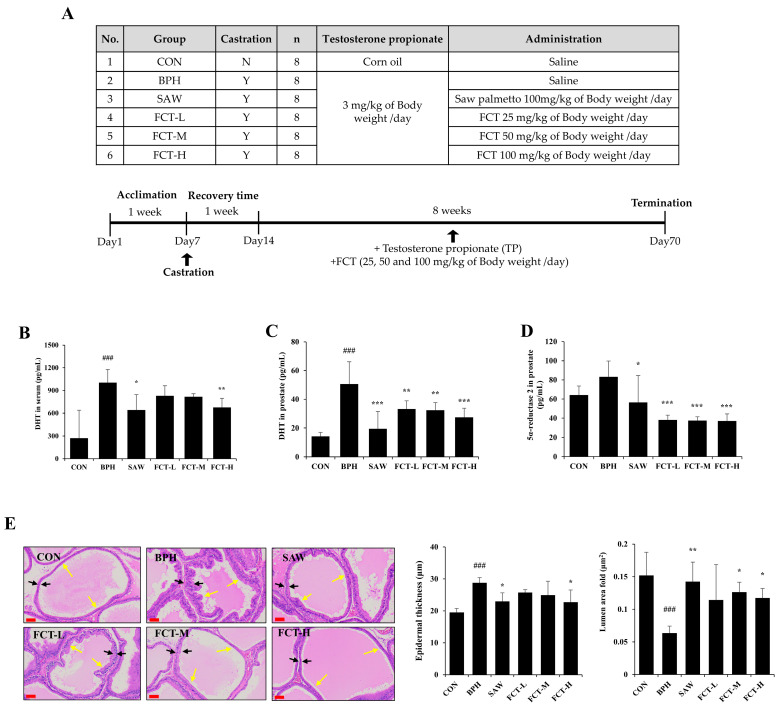
Effects of FCT on prostate weight, DHT levels, 5α-reductase 2 expression, and prostate H&E staining in BPH-induced rats. (**A**) Description of experimental animal groups and study design. (**B**) Serum DHT levels, (**C**) prostate DHT levels and (**D**) prostate 5α-reductase 2 were measured using an ELISA assay. (**E**) H&E staining analyses were performed on prostate tissue (magnification 20×, scale bar 60 µm). Black arrows indicate the epithelial layer and yellow arrows mark the lumen area. CON: normal control group; BPH: 3 mg/kg TP injection; SAW: saw palmetto 100 mg/kg + 3 mg/kg TP injection; FCT-L: FCT 25 mg/kg + 3 mg/kg TP injection; FCT-M: FCT 50 mg/kg + 3 mg/kg TP injection; FCT-H: FCT 100 mg/kg + 3 mg/kg TP injection. Data are presented as mean ± standard deviation (SD). Significant differences between CON and BPH group were denoted by ### *p* < 0.001, and versus the BPH group was denoted by * *p* < 0.05, ** *p* < 0.01, and *** *p* < 0.001, respectively.

The serum DHT level in the BPH group increased by approximately 2.7 times compared to the CON group (Figure 2B). The FCT-H groups had significant reductions in serum DHT levels, by 32.78%, which was higher than that of the SAW group (*p* < 0.01). Prostate DHT levels in the BPH group were 2.5 times higher than those in the CON group; however, FCT significantly reduced prostate DHT levels in all groups. (Figure 2C). In addition, the prostate DHT levels in the SAW group significantly decreased compared to the BPH group (*p* < 0.01).

Prostate 5α-reductase 2 levels (Figure 2D), increased by approximately 29.43% in the BPH group compared to the CON group (no significant difference). All FCT-treated groups experienced significant reductions in 5α-reductase 2 levels compared to the BPH group (*p* < 0.001), with reductions greater than those observed in the SAW group.

Figure 2E shows the results of H&E staining of prostate tissue to examine the histological changes induced by FCT. In the BPH group, compared to the CON group, epithelial thickness was significantly increased due to epithelial cells protruding into the glandular lumen and glands surrounded by double-layered epithelium, while the glandular lumen size was reduced (*p* < 0.05). In contrast, the high-dose FCT-treated group showed a significant reduction in epithelial thickness, by approximately 21.03%, compared to the BPH group and exhibited a structure with thin-walled tubular acini, similar to that of the CON group (*p* < 0.05). The SAW group also showed a reduction in epithelial thickness by approximately 20.26% compared to the BPH group, reaching a level similar to that of the FCT-L group. Furthermore, the lumen area in the FCT-M and FCT-L groups significantly increased by 77.90% and 65.20%, respectively, compared to the BPH group, demonstrating a marked increase in glandular lumen area and a reduction in the height of the glandular epithelium (*p* < 0.05). The SAW group also showed an increase in lumen area compared to the BPH group (*p* < 0.05).

### 2.3. FCT Regulated Apoptosis Signaling via Androgen Receptor in BPH-Induced Rats

**Figure 3:**
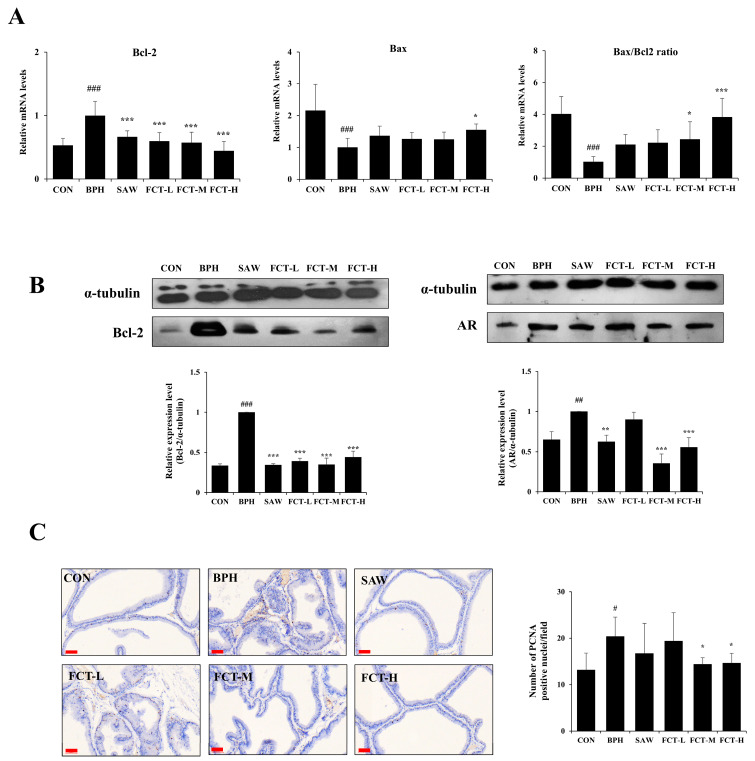
Effects of FCT on Bcl-2, Bax, AR, and PCNA expression in BPH-induced rats. (**A**) The mRNA expression levels of the Bcl-2, Bax and Bax/Bcl-2 ratio were measured by qRT-PCR with GAPDH as the internal control. (**B**) Bcl-2 and AR protein expression were detected by Western blot analysis, using α-tubulin as the loading control. (**C**) PCNA expression was identified using immunohistochemical (IHC) staining of prostate tissue (magnification 20×, scale bar 60 µm). CON: normal control group; BPH: 3 mg/kg TP injection; SAW: saw palmetto 100 mg/kg + 3 mg/kg TP injection; FCT-L: FCT 25 mg/kg + 3 mg/kg TP injection; FCT-M: FCT 50 mg/kg + 3 mg/kg TP injection; FCT-H: FCT 100 mg/kg + 3 mg/kg TP injection. Data are presented as mean ± standard deviation (SD). Significant differences between CON and BPH group are denoted by # *p* < 0.05, ## *p* < 0.01, and ### *p* < 0.001, and, compared to the BPH group, denoted by * *p* < 0.05, ** *p* < 0.01, and *** *p* < 0.001, respectively.

Bcl-2 mRNA expression in the prostate tissue increased by 87.98% in the BPH group compared to that in the control group (Figure 3A). However, it was significantly decreased in the FCT-treated groups, similar to that in the SAW group. In addition, the expression of Bcl-2 protein levels was significantly reduced in the FCT group compared to that in the BPH group (Figure 3B). Bax mRNA expression decreased by 53.67% in the BPH group but increased by 55.10% in the FCT-H group compared to the BPH group, exceeding the increase observed in the SAW group. The Bax/Bcl-2 ratio in the BPH group decreased by 74.49% compared to the CON group. However, it significantly increased in FCT-L and FCT-H groups, with the FCT-H group showing an approximate 2.7-fold increase, 1.5 times higher than that of the SAW group (*p* < 0.001). Next, AR protein levels increased more in the BPH group compared to those in the CON group; however, they decreased in the FCT-M, FCT-H, and SAW groups (Figure 3B).

PCNA-positive nuclei in the prostate tissue were markedly increased in the BPH group compared to those in the CON group (*p* < 0.05) (Figure 3C). In addition, PCNA-positive cells significantly decreased in the FCT-M and FCT-H groups (29.21 and 28.10%, respectively) (*p* < 0.05).

### 2.4. FCT Reduced the mRNA Expression of NF-κB and Pro-Inflammatory Cytokines in BPH-Induced Rats

**Figure 4:**
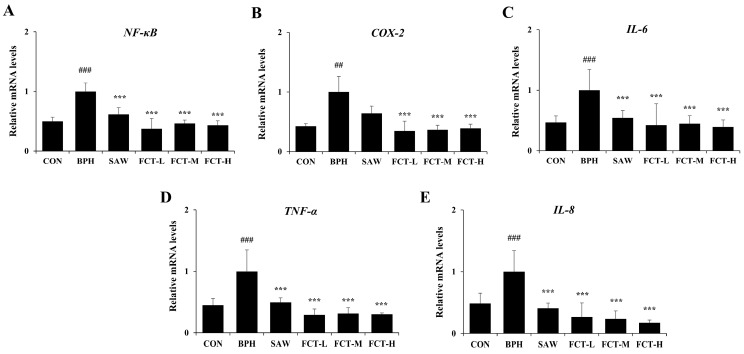
Effects of FCT on pro-inflammatory cytokines in BPH-induced rats. mRNA expression levels of (**A**) NF-κB, (**B**) COX-2, (**C**) IL-6, (**D**) TNF-α, and (**E**) IL-8 were quantified by qRT-PCR. GAPDH was used for data normalization. CON: normal control group, BPH: 3 mg/kg TP injection, SAW: saw palmetto 100 mg/kg + 3 mg/kg TP injection, FCT-L: FCT 25 mg/kg + 3 mg/kg TP injection, FCT-M: FCT 50 mg/kg + 3 mg/kg TP injection, FCT-H: FCT 100 mg/kg + 3 mg/kg TP injection. Data are presented as mean ± standard deviation (SD). Significant differences between CON and BPH group were denoted by ## *p* < 0.01, and ### *p* < 0.001, and versus the BPH group was denoted by *** *p* < 0.001, respectively.

The mRNA levels of pro-inflammatory cytokines were examined to assess changes in the inflammatory response in rats with TP-induced BPH (Figure 4). The mRNA expressions of NF-κB, COX-2, TNF-α, IL-6, and IL-8 were significantly increased in the BPH group compared to the normal group; however, FCT administration reduced their expression. The NF-κB mRNA expression levels were reduced above 50% compared to the BPH group following FCT treatment (*p* < 0.001) (Figure 4A). COX-2 expression decreased by more than 60% in the FCT-treated groups (*p* < 0.001) (Figure 4B). In addition, FCT treatment led to a decrease in the expression levels of TNF-α, IL-6, and IL-8 in all groups (*p* < 0.001), with significant reductions observed in the SAW group. (Figure 4C–E). Notably, IL-8 expression decreased by more than 80% in the FCT-H group (*p* < 0.001).

### 2.5. FCT Inhibited Cell Proliferation and Decreased the mRNA Expression Levels of AR, Steroid 5 Alpha-Reductase 2 (SRD5A2), and Bcl-2 in BPH-1 Cells

**Figure 5:**
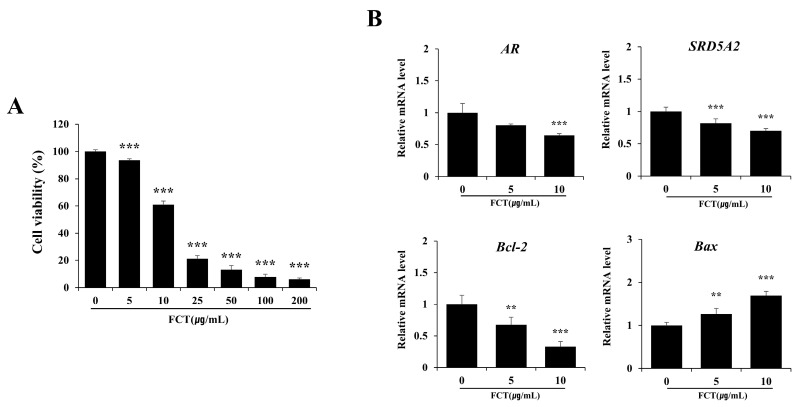
Effects of FCT on cell proliferation and mRNA expression levels of AR, SRD5A2, Bcl-2, and Bax in BPH-1 cells. (**A**) Cell viability was measured in BPH-1 cells after FCT treatment (0 to 200 µg/mL) for 24 h using the MTT assay. (**B**) mRNA expression levels of AR, SRD5A2, Bcl-2, and Bax were analyzed after FCT treatment (0 to 10 µg/mL) for 24 h by qRT-PCR, using GAPDH as the control. Data are presented as mean ± standard deviation (SD). Significant differences relative to the control were denoted by ** *p* < 0.01 and *** *p* < 0.001, respectively.

We examined the changes in cell proliferation in BPH-1 epithelial cells following FCT treatment (Figure 5A). A significant reduction in cell viability was observed at a concentration of 5 µg/mL of the extract, with decreases of 6.37, 39.03, and 78.79% at concentrations of 5, 10, and 25 µg/mL, respectively, showing a dose-dependent decrease. Cell viability decreased by over 80% at concentrations of 50 µg/mL or higher, indicating a strong inhibitory effect on cell proliferation due to FCT (*p* < 0.001).

In addition to the inhibition of cell proliferation, we investigated changes in the mRNA expression levels of AR, SRD5A2, Bcl-2, and Bax, which are involved in the regulation of apoptosis, in the FCT-treated groups (Figure 5B). AR expression significantly decreased by 35.52% following treatment with FCT (10 µg/mL), whereas SRD5A2 showed a dose-dependent decrease starting from 5 µg/mL. Bcl-2 expression significantly decreased, by 66.80%, at 10 µg/mL. In contrast, Bax expression increased in a dose-dependent manner from 5 to 10 µg/mL.

### 2.6. FCT Suppressed the Protein Expression of NF-κB p65, COX-2, TNF-A, IL-6, and IL-1β in Cells

**Figure 6:**
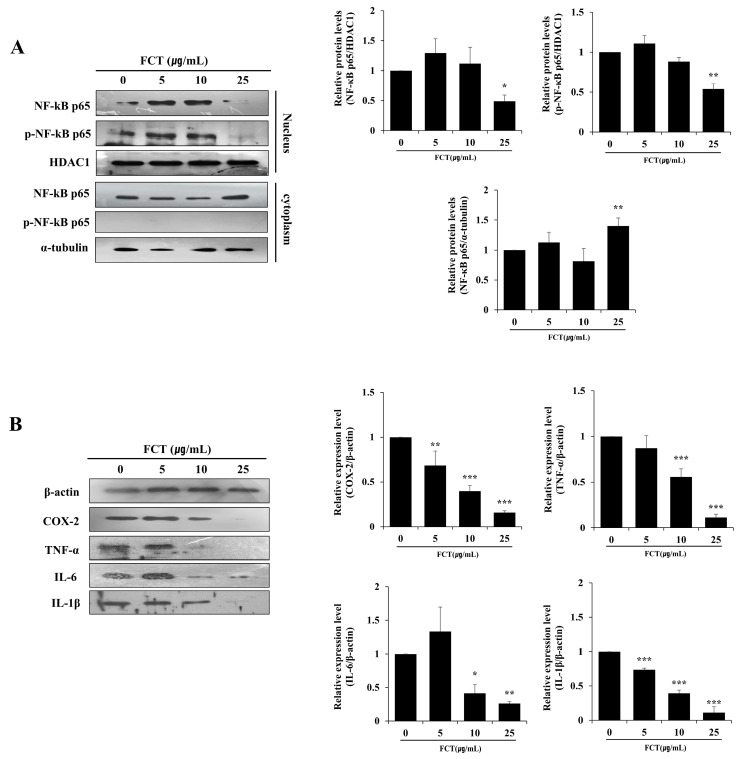
Effects of FCT on NF-kB p65 and pro-inflammatory cytokines’ protein expression in BPH-1 cells. (**A**) Western blot analysis was performed to detect nucleus/cytoplasm NF-kB p65 protein levels in BPH-1 cells. HDAC1 in the nucleus and α-tubulin in the cytoplasm were used as loading controls. (**B**) Pro-inflammatory cytokine protein levels were measured by Western blot analysis, with β-actin as the internal control. Data are presented as mean ± standard deviation (SD). Significant differences versus control were denoted by * *p* < 0.05, ** *p* < 0.01, and *** *p* < 0.001, respectively.

Treatment of BPH-1 cells with FCT resulted in a dose-dependent reduction in NF-κB p65 expression (Figure 6A). FCT at 25 µg/mL reduced nuclear translocation of NF-κB p65 and decreased phosphorylated NF-κB p65 levels within the nucleus. Consequently, FCT suppressed NF-κB p65 activation, alongside a decrease in pro-inflammatory cytokine expression.

We examined the protein expression changes in COX-2, TNF-α, IL-6, and IL-1β to investigate the relationship between FCT and inflammatory responses in BPH-1 cells (Figure 6B). FCT at 5 µg/mL did not affect the expression of TNF-α and IL-6. However, we observed a decrease in protein expression at 10 µg/mL. Notably, at 25 µg/mL, the expression of COX-2, TNF-α, IL-6, and IL-1β were significantly reduced.

## 3. Discussion

BPH has become a major urological health problem that reduces the quality of life of men increasingly with the age of the population [22]. The pathophysiology of BPH is poorly understood; however, hormones, imbalances between prostate proliferation and apoptosis, and inflammation are reportedly associated with the development and progression of the disease [23,24].

In the prostate gland, the more potent androgen, DHT, is synthesized from testosterone and catalyzed by 5AR. DHT has a three-fold higher affinity for AR compared to testosterone, mediating BPH development [25]. In addition, AR expression was significantly higher in patients with BPH than in normal prostate tissue, as observed in both epithelial and stromal cells [10]. In this study, FCT-treated groups (FCT-M and -H) showed reduced prostate weight, DHT levels in both serum and prostate tissue, and reduced 5AR and AR expression levels in a testosterone-propionate induced BPH animal model.

An imbalance between cell proliferation and death is a prominent factor in BPH. The anti-apoptotic gene Bcl-2 was significantly upregulated, whereas Bax, p53, and caspase-3 were significantly downregulated in the BPH-induced rat model [10,24]. Various studies reported that the expression of NF-κB was enhanced in the BPH model, and it affects the cell proliferation/apoptosis balance, including Bcl-2 upregulation and inflammation. Specifically, NF-κB was activated in the BPH-induced group, and the expression of IL-8, TNF-α, and COX-2 increased significantly [24,26]. In this study, the FCT-treated group experienced downregulated Bcl-2 expression, upregulated relative expression of Bax/Bcl-2, and reduced expression of PCNA (the proliferation marker). In addition, the FCT-treated group had reduced NF-κB and inflammatory factors, comprising COX-2, TNF-α, IL-6, and IL-8, compared to the BPH group.

NF-κB is a transcription factor that regulates genes involved in cell proliferation, apoptosis, inflammation, and other biological processes [27,28]. The transcription factor NF-κB, activated by various stimuli including cytokines and growth factors, translocates from the cytoplasm to the nucleus to initiate transcriptional responses, upregulating genes mediating cell proliferation such as TNF-α, IL-6, and IL-1β upon appropriate stimulation [29,30]. Inflammation is important in BPH, and TNF-α, IL-6, and IL-8 are major pro-inflammatory cytokines involved in the occurrence and development of BPH [23,26]. IL-1β is a key cytokine in regulating the inflammatory process and inducing COX-2 expression [27]. COX-2 is upregulated in the prostate during significant inflammation and is associated with Bcl-2 overexpression in BPH [31]. In this study, NF-κB was activated by its translocation from the cytosol to the nucleus of BPH-1 cells. However, following FCT treatment of BPH-1 cells, nuclear translocation of NF-κB was suppressed, resulting in the downregulation of genes associated with cell proliferation and inflammation. Moreover, FCT treatment of BPH cells suppressed pro-inflammatory cytokines, which is consistent with the in vivo results.

Fenugreek seed contains alkaloids, flavonoids, and oils, primarily linolenic acid, linoleic acid, and oleic acid [14]. Vitexin was used as the standard compound in high-performance liquid chromatography (HPLC) analysis for FCT. The key distinguishing factor of the fenugreek extract used in this study compared to conventional fenugreek extracts (Testofen) lies in the composition and content of polyphenolic compounds. The vitexin content of the fenugreek extract used in this experiment is approximately 3–4 times higher than that of the extract in previous studies. Structural differences in polyphenolic compounds result in changes in physical and chemical properties, and bioactivity, which may lead to different effects between the two extracts [32]. Vitexin is a polyphenolic compound known for its prominent anti-inflammatory properties by modulating the NF-κB pathway and pro-inflammatory cytokines [33,34]. A previous study reported that the fenugreek seed extract reduced IL-6 in an ovariectomized rat model [35] and downregulated NF-κB, TNF-α, IL-6, IL-8, and IL-1β in a pulmonary fibrosis animal model [36]. In the current study, the effect of FCT on BPH is attributed to its various bioactive compounds, which modulate the NF-κB pathway and pro-inflammatory cytokines. We hypothesize that the effects of FCT on BPH are attributed to vitexin and its anti-inflammatory and anti-proliferative properties.

In this study, we showed that FCT includes flavone-c-glycoside such as vitexin, iso-vitexin, orientin, and iso-orientin through HPLC analysis. The flavone-c-glycoside generates aglycones, such as deglycosylated and acetylated metabolites, in the intestine through the metabolic pathways like glucuronidation and sulfation [37]. Dong’s study reported that vitexin, when absorbed and fragmented in vivo, generates intermediates such as apigeni, and fragment ions with high abundance [38]. Additionally, Shi’s study found that orientin is metabolized in the human intestine, releasing aglycones, and specific strains generate luteolin [37].

The structural diversities of flavonoid compounds largely contribute to their physical, chemical properties, and antioxidant activity [32]. Therefore, it is important to note that the effective forms of C-glycoside flavonoids such as vitexin and orientin may be the metabolites, like flavonols or aglycones, arising from in vivo biotransformation. In contrast, flavonoids exhibit high antioxidant activity in vitro, which may not be the case in vivo [32]. Thus, our further study will focus on examining the changes in physiological activity when the metabolites of FCT, including vitexin, are absorbed in vivo.

We observed a discrepancy between the in vitro and in vivo results with FCT treatment. For example, in the in vitro study, the apoptosis-related genes Bax and Bcl-2, as well as anti-inflammatory cytokines, were dose-dependently downregulated following FCT treatment. However, in the in vivo study, their downregulation did not exhibit a dose-dependent pattern. In a previous report [39,40], flavone C-glycosides were found to have poor absorption in the rat intestine and to potentially resist degradation by gut bacteria in rats, as less than 1% was excreted in urine and 10–88% was recovered from feces. Although the efficacy of flavonoids, including flavone C-glycoside, in animal studies is not easy to predicted based on in vitro results, the activity of flavone could be explained in the intestinal lumen rather than in circulation [39].

This discrepancy may be attributed to the bioavailability of flavonoids and the complexity of their metabolic pathways. In future studies, the analysis of flavonoids in blood, urine, and feces will be necessary to clarify the underlying causes of these inconsistencies between in vitro and in vivo results. We plan to investigate the effects of vitexin and orientin, including intermediates like apigenin, luteolin, and fragment ions on BPH and clarify their mechanisms of action.

## 4. Materials and Methods

### 4.1. Preparation of Fenugreek Seed Extracts

We obtained the FCT powder (Fenugreek seed ethanol extract) from Daehan Chemtech Co., Ltd. (Gwacheon, Republic of Korea). The fenugreek seed was prepared through hydroalcoholic extraction. It was first extracted with a solvent mixture of ethanol and water (80:20 *v*/*v*) for 8 h. The mixture was then filtered and concentrated. The concentrated product was dried, sieved, and blended. FCT was standardized to have a vitexin content of 3–4% and was manufactured in a GMP-compliant facility.

### 4.2. Liquid Chromatography

FCT powder was accurately weighed in a volumetric flask for HPLC analysis. Subsequently, ethanol (80%) was added to the flask, and the contents were sonicated for 10 min. We performed the analysis at 30 °C using a LiChrosorb^®^ RP18 column (4.6 × 250 mm, 5 µm particle size) and at a wavelength of 330 nm. The mobile phases were solvent A (0.1% orthophosphoric acid in water) and B (acetonitrile). Afterward, the analysis was performed at a flow rate of 1.5 mL/min under the following linear gradient conditions: 90% A to 10% B for 8 min; 87% A to 13% B for 17 min; 80% A to 20% B for 5 min; 80% A to 20% B for 10 min; 0% A to 100% B for 5 min; and 90% A to 10% B for 2 min.

### 4.3. Cell Proliferation

We cultured BPH-1, a human benign prostate hyperplastic cell line, in RPMI 1640 medium containing 20% fetal bovine serum, 1% antibiotics, 10 ng/mL insulin–transferrin–sodium selenite, and 20 ng/mL testosterone. The method for cell viability measurement followed a previous study [41]. Subsequently, we seeded a 96-well plate with 10,000 cells/well, added 0 to 200 μg/mL of FCT, and cultured for 24 h. Afterwards, 5 mg/mL of 3-(4,5-Dimethylthiazol-2-yl)-2,5-diphenyltetrazolium bromide was added. The absorbance was measured after 2 h by subtracting the absorbance at 630 nm from that at 570 nm. Cell proliferation was calculated considering the control as 100%.

### 4.4. Separation of the Nuclear and the Cytoplasmic Fractions in BPH-1 Cells

Cells were seeded at a density of 10^6^ cells/mL in a 100 mm dish and incubated for 24 h. After treating the cells with FCT at concentrations of 0, 5, 10, and 25 µg/mL for 24 h, the cells were centrifuged at 500× *g* for 2–3 min to collect the pellet. Nuclear and cytoplasmic fractions were separated from the cells using NE-PER™ Nuclear and Cytoplasmic Extraction Reagents (Thermo Fisher Scientific, Waltham, MA, USA) according to the manufacturer’s instructions. The separated fractions were then subjected to lysis for subsequent Western blot analysis.

### 4.5. Quantitative Reverse Transcription Polymerase Chain Reaction (qRT-PCR)

We used RNA-iso Plus (Takara, Shiga, Japan) for RNA extraction according to the manufacturer’s protocol. Complementary DNA was synthesized from RNA using reverse transcriptase reagent (Takara), a T100 Thermal Cycler (Bio-Rad, Hercules, CA, USA). qRT-PCR was performed using a Light Cycler 96 (Roche, Basel, Switzerland) and FastStart Essential DNAS Green Master Mix (Roche, Basel, Switzerland). The samples were initially preincubated at 95 °C for 10 min, followed by 40 amplification cycles under the following conditions: denaturation at 95 °C for 10 s, annealing at 55 °C for 10 s, and extension at 72 °C for 10 s. This was succeeded by a final step of 95 °C for 5 s and 65 °C for 60 s. The cooling step was carried out at 37 °C for 30 s. All experiments were performed in triplicate. The primer sequences are listed in Supplemental Table S1.

### 4.6. Animals

We acclimatized seven-week-old male Sprague Dawley rats (*n* = 48; Saeron Bio, Uiwang, Republic of Korea) for one week under controlled conditions at 23 ± 2 °C, 55 ± 5% relative humidity, and a 12 h light–dark cycle. The study protocol was approved by the Institutional Animal Care and Utilization Committee of Suwon University (approval code and date: USW-IACUC-2023-001 and 13 April 2023). Subsequently, castration was performed in all groups, excluding the control group (*n* = 8). Testosterone propionate (TP) (3 mg/kg body weight/day) was injected subcutaneously to induce prostate hypertrophy after stabilization for a week. The above experiment was conducted for each animal group as follows: Water and food were freely consumed throughout the experiment, and the samples were administered orally daily for eight weeks. The rats were used and randomly divided into the following 6 groups: (A) injected with corn oil subcutaneously and treated with saline by oral gavage (CON; *n* = 8); (B) injected with TP (3 mg/kg B.W/day) and treated with saline (BPH; *n* = 8); (C) injected with TP (3 mg/kg B.W/day) and treated with FCT (25 mg/kg B.W/day) (FCT-L; *n* = 8); (D) injected with TP (3 mg/kg B.W/day) and treated with FCT (50 mg/kg B.W/day) (FCT-M; *n* = 8), (E) injected with TP (3 mg/kg B.W/day) and treated with FCT (100 mg/kg B.W/day) (FCT-H; *n* = 8); (F) injected with TP (3 mg/kg B.W/day) and treated with saw palmetto (100 mg/kg B.W/day) (SAW; *n* = 8). After eight weeks, all animals were sacrificed using Carbon dioxide (CO_2_) anesthesia. Blood was collected via cardiac puncture and centrifuged at 1500× *g* for 20 min to obtain serum. To isolate the prostate from the abdomen, the seminal vesicles, bladder, and prostate were separated sequentially. Using fine scissors, the bladder was removed first, followed by the seminal vesicles, leaving only the prostate surrounding the urethra. Prostate tissues were weighed, and organs were stored at −80 °C.

### 4.7. Castration

Castration was performed based on the OECD Hershberger Bioassay [42]. The scrotal skin and peritoneum of the animal are incised to expose the testes, and the blood vessels connecting to the vas deferens are ligated with surgical sutures. The testes are removed, and the open scrotal skin is closed by applying automatic clips to the incision edges. After a 7-day recovery period following the surgery, the administration of FCT and TP injection begins.

### 4.8. H&E Staining

The ventral and lateral prostate tissues were fixed in 4% formaldehyde and embedded in melting wax to prepare the paraffin blocks. The prostate tissue staining method followed a previous study [41]. The paraffin sections were dewaxed in xylene solution and dehydrated in ethanol for staining. The tissue was dehydrated, followed by eosin staining, and finally covered with coverslips after staining with hematoxylin.

### 4.9. Immunohistochemistry (IHC)

Prostate tissues were embedded in paraffin blocks for proliferating cell nuclear anti-gen antibody staining after fixation with 4% formaldehyde. The overall method for IHC staining followed a previous study [43]. Each section was dehydrated by washing with xylene and ethanol. Subsequently, the sections were incubated with 3% hydrogen peroxide and washed for blocking. The primary antibody was incubated overnight, and after washing with the wash buffer, the secondary antibody was incubated for 30 min. The sections were incubated using ABC (Avidin/Biotin Complex), and the staining results were visualized using a DAB kit as a substrate. Afterward, the sections were dehydrated again and mounted on coverslips.

### 4.10. Western Blot Analysis

The cells were lysed in Radioimmunoprecipitation Assay (RIPA) buffer and centrifuged at 13,000× *g* for 15 min to obtain the supernatant. Similarly, prostate tissue was homogenized in RIPA buffer and centrifuged for protein quantification. The method and procedure for Western blot analysis followed a previous study [41]. The protein samples (15 µg) were separated by sodium dodecyl sulfate–polyacrylamide gel electrophoresis (SDS-PAGE) gels and transferred onto nitrocellulose membranes. The membranes were blocked with 5% skim milk and incubated overnight at 4 °C with primary antibodies. Furthermore, the membranes were incubated with horseradish peroxidase (HRP)-conjugated secondary antibodies. Signals were detected using an enhanced chemiluminescence (ECL) substrate and an X-ray film. The primary and secondary antibodies used are as follows: AR (Thermo Fisher Scientific, Waltham, MA, USA), Nuclear factor-kappa B, tumor necrosis factor-alpha, Histone Deacetylase, interleukin-6, and interleukin-1β (Santa Cruz Biotechnology, Dallas, TX, USA, respectively), phosphorylated NF-κB (Cell Signaling Technology, Danvers, MA, USA), α-tubulin (Merk Millipore, Burlington, MA, USA), Cyclooxygenase-2 (Cell Signaling Technology). Anti-rabbit IgG and anti-mouse IgG (Cell Signaling Technology) were used. All antibodies were diluted at a ratio of 1:1000 in 5% skim milk.

### 4.11. Chemical Analysis of Serum

The NX500i (Fujifilm, Tokyo, Japan) was used to analyze serum samples obtained by centrifuging blood from experimental animals at 1500× *g* for 30 min. Alanine aminotransferase (ALT) levels and aspartate aminotransferase (AST) levels were measured. The detailed protocol for serum analysis followed a previous study [41].

### 4.12. ELISA in Serum and Prostate

DHT levels were assessed using a DHT ELISA assay kit (Cusabio, Houston, TX, USA) in both the serum and prostate. ELISA analysis was performed following the manufacturer’s instructions, and the detailed methods were referenced from a previous study [41]. Prostate tissues were homogenized in phosphate-buffered saline and incubated at −20 °C overnight. The samples were centrifuged at 4 °C and 5000 rpm for 5 min, and the supernatant was collected for analysis after two freeze–thaw cycles were performed to damage the cell membrane. The supernatant and serum were incubated with HRP-conjugated DHT and DHT-specific antibody in a 96-well microplate pre-coated with goat-anti-rabbit antibody at 37 °C for 1 h. Finally, 5-alpha reductase 2 activity in the prostate samples was evaluated using the 5-alpha reductase 2 ELISA kit (Cusabio, Houston, TX, USA).

### 4.13. Statistical Analysis

The experimental groups in this experiment were expressed as mean ± SD. Statistical significance was assessed using one-way analysis of variance (ANOVA), followed by Dunnett’s post hoc test after confirming normality assumptions. The Kruskal–Wallis H test was performed for nonparametric analysis, followed by Dunn’s post hoc test. Statistical significance was set at *p* < 0.05. The Statistical Package for Social Science, version 22.0 (IBM Co., Armonk, NY, USA) was used for statistical analysis.

## 5. Conclusions

In summary, our results showed that FCT treatment reduced DHT and 5-alpha reductase levels, prostate weight in the BPH-rat model, and regulated cell proliferation and apoptosis through modulating NF-kB and pro-inflammatory cytokines, including TNF-α, IL-6, IL-8, and IL-1β. These suggest that FCT could ameliorate BPH.

## Figures and Tables

**Table 1:** The effects of FCT on prostate in TP-induced BPH rats.

Group	Prostate Weight (g)	Prostate Ratio ^1^
CON	0.66 ± 0.15	0.16 ± 0.03
BPH	1.23 ± 0.18 ###	0.33 ± 0.05 ###
SAW	1.00 ± 0.15 *	0.24 ± 0.05 **
FCT-L	1.05 ± 0.13	0.28 ± 0.05
FCT-M	0.96 ± 0.18 *	0.26 ± 0.06 *
FCT-H	0.91 ± 0.22 **	0.24 ± 0.06 **

^1^ Prostate ratio: prostate weight (mg)/body weight (g) × 100. CON: normal control group; BPH: 3 mg/kg TP injection; SAW: saw palmetto 100 mg/kg + 3 mg/kg TP injection; FCT-L: FCT 25 mg/kg + 3 mg/kg TP injection; FCT-M: FCT 50 mg/kg + 3 mg/kg TP injection; FCT-H: FCT 100 mg/kg + 3 mg/kg TP injection. Values are presented as means ± SD. Significant differences between CON and BPH group were denoted by ### *p* < 0.001, and those versus the BPH group were denoted by * *p* < 0.05 and ** *p* < 0.01, respectively.

## Data Availability

The original contributions presented in this study are included in the article/Supplementary Materials. Further inquiries can be directed to the corresponding author.

## Supplementary Materials

The following supporting information can be downloaded at https://www.mdpi.com/article/10.3390/ijms26031261/s1.

## Abbreviations

The following abbreviations are used in this manuscript:

BPH	Benign Prostatic Hyperplasia
DHT	Dihydrotestosterone
T	Testosterone
TP	Testosterone Propionate
SD	Sprague–Dawley
FCT	Fenugreek seed extract (Forceterone^®^)
COX-2	Cyclooxygenase-2
TNF-α	Tumor Necrosis Factor-α
IL-1β	Interleukin-1β
IL-6	Interleukin-6
IL-8	Interleukin-8
Bcl-2	B-cell lymphoma 2
Bax	Bcl-2 associated protein X
NF-κB	Nuclear factor κB
pNF-κB	Phosphorylated NF-κB
PCNA	Proliferating Cell Nuclear Antigen
AR	Androgen Receptor
