# Supplementary material for: Effect of Fenugreek Extract on Testosterone Propionate-Induced Benign Prostatic Hyperplasia
Source: Int J Mol Sci. 2025 Jan 31;26(3):1261. doi: 10.3390/ijms26031261 (PMC11818512; doi:10.3390/ijms26031261)
Supplement: Supplementary file 1 [file ijms-26-01261-s001.zip › ijms-3421416-supplementary.pdf]

Table S1. Primer sequences for quantitative real time PCR.

Gene	Forward (5′→3′)	Reverse (5′→3′)
Human		
GAPDH	GTCCACCACCTGTTGCTGTAG	CAAGGTCATCCATGACAACTTTG
AR	CAGTGGATGGGCTGAAAAAT	AGGAGCTTGGTGAGCTGGTA
SRD5A2	CTACGGGAAGCACACGGAGAGCCTGAA	CCAGGCTTCCTGAGCTGGCGCAATATATAG
Bcl-2	ATGTGTGTGGAGAGCGTCAA	ACAGTTCCACAAAGGCATCC
BAX	TTGCTTCAGGGTTTCATCCA	CAGCCTTGAGCACCAGTTTG
Rat		
GAPDH	CAACTCCCTCAAGATTGTCAGCAA	GGCATGGACTGTGGTCATGA
Bcl-2	GCCGGTTCAGGTACTCAGTCAT	AGGATTGTGGCCTTCTTTGAGT
BAX	GATCAGCTCGGGCACTTT	TGTTTGCTGATGGCAACTTC
IL-6	CTGCAAGAGACTTCCATCCAG	AGTGGTATAGACAGGTCTGTTGG
IL-8	CATTAATATTTAACGATGTGGATGCGTTTCA	GCCTACCATCTTTAAACTGCACAAT
TNF-α	CCCTGGTACTAACTCCCAGAAA	TGTATGAGAGGGACGGAACC
NF-κB	ATCAATGGCTACACGGGACC	AGTTCATGTGGATGAGGCCG
COX-2	ATAAGTGCGATTGTACCCG	TAGCCATAGTCAGCATTGTAAGT

Table S2. Effect of FCT on body weight and FER in BPH-induced rats.

Group	Initial BW (g)	Final BW (g)	BW gain (g) [1]	FER [2]
CON	252.65 ± 16.43	411.26 ± 20.26	158.61 ± 24.27	0.20 ± 0.03
BPH	246.48 ± 7.30	387.24 ± 30.88	140.77 ± 28.68	0.21 ± 0.05
SAW	254.91 ± 8.31	403.73 ± 23.53	148.82 ± 19.21	0.21 ± 0.02
FCT-L	245.89 ± 7.90	386.78 ± 21.12	140.90 ± 22.62	0.19 ± 0.03
FCT-M	255.82 ± 15.30	385.36 ± 25.36	129.54 ± 36.52	0.18 ± 0.05
FCT-H	254.40 ± 10.88	379.40 ± 18.25	125.01 ± 18.81	0.18 ± 0.03

[1] Body weight gain (g) = final body weight (g) - initial body weight (g). [2] FER (food efficiency ratio) = body weight gain (g) / total food intake (g). Values are presented as means ± SD. Significant differences between CON and BPH group were denoted by # $p < 0.05$, and versus the BPH group was denoted by * $p < 0.05$, respectively. There are no significant differences among the groups.

Table S3. Effect of FCT on liver damage in BPH-induced rats.

Group	Liver index [1]	ALT	AST
CON	3.57 ± 0.22	37.20 ± 7.19	83.00 ± 16.43
BPH	3.57 ± 0.49	32.71 ± 7.30	72.00 ± 23.24
SAW	3.49 ± 0.12	34.29 ± 21.59	71.63 ± 21.67
FCT-L	3.51 ± 0.20	36.17 ± 18.43	86.60 ± 26.86
FCT-M	3.38 ± 0.19	40.00 ± 14.39	82.83 ± 16.40
FCT-H	3.39 ± 0.22	31.57 ± 5.16	69.83 ± 14.08

[1] Liver index = Liver weight (g) / Body weight (g) × 100. Values are presented as means ± SD. Significant differences between CON and BPH group were denoted by # $p < 0.05$, and versus the BPH group was denoted by * $p < 0.05$, respectively. There are no significant differences among the groups.
